# Potentially inappropriate prescribing in Ethiopian geriatric patients hospitalized with cardiovascular disorders using START/STOPP criteria

**DOI:** 10.1371/journal.pone.0195949

**Published:** 2018-05-03

**Authors:** Tadesse Melaku Abegaz, Eshetie Melese Birru, Gashaw Binega Mekonnen

**Affiliations:** 1 Department of clinical pharmacy, school of pharmacy, college of medicine and health sciences, university of Gondar, Gondar, Ethiopia; 2 Department of pharmacology, school of pharmacy, college of medicine and health sciences, university of Gondar, Gondar, Ethiopia; University of Brescia, ITALY

## Abstract

**Background:**

There was a paucity of data on the magnitude of potentially inappropriate prescriptions (PIPs) among Ethiopian elderly cardiovascular patients.

**Objective:**

The aim of this study was to assess PIPs and associated factors in the elderly population with cardiovascular disorders using the START/STOPP screening criteria.

**Methods:**

A hospital-based cross-sectional study was conducted at medical wards of a teaching hospital in Ethiopia from 1 December 2016–30 May 2017. Included patients were hospitalized elderly patients aged 65 years or older with cardiovascular disorders; their medications were evaluated using the START/STOPP screening criteria from admission to discharge. Multivariable logistic regression was applied to identify factors associated with inappropriate medications. One Way Analysis Of Variance (ANOVA) was carried out to test significant differences on the number of PIPs per individual diagnosis.

**Results:**

Two hundred thirty-nine patients were included in the analysis. More-than a third of the patients were diagnosed with heart failure, 88 (36.82%). A total of 221 PIPs were identified in 147 patients, resulting in PIP prevalence of 61.5% in the elderly population. Of the total number of PIPs, occurrence of one, two and three PIPs accounted for 83 (56.4%), 52(35.4%), and 12(8.2%) respectively. One way ANOVA test showed significant differences on the mean number of PIPs per individual diagnosis (f = 5.718, p<0.001). Angiotensin Converting Enzyme Inhibitors (ACEIs) were the most common inappropriately prescribed medications, 32(14.5%). Hospital stay, AOR: 1.086 (1.016–1.160), number of medications at discharge, AOR: 1.924 (1.217–3.041) and the presence of co-morbidities, AOR: 3.127 (1.706–5.733) increased the likelihood of PIP.

**Conclusion:**

Approximately, two-thirds of elderly cardiovascular patients encountered potentially inappropriate prescriptions. ACEIs were the most commonly mis-prescribed medications. Longer hospital stay, presence of comorbidities and prescription of large number of medications at discharge have been correlated with the occurrence of inappropriate medication. It is essential to evaluate patients’ medications during hospital stay using the STOPP and START tool to reduce PIPs.

## Introduction

Potentially inappropriate prescribing (PIP) encompasses problems of prescribing potentially inappropriate medications (PIMs) and prescription omissions (PPOs). The risks of the PIMs outweighs their benefits among older people [1-2]. Screening tool of older people’s (age≥65) prescription (STOPP) and screening tool to alert physicians for the right therapy (START) criteria are special medication prescribing tools employed to evaluate medication appropriateness by preventing and avoiding adverse drug reactions (ADRs), polypharmacy and medication omissions [2]. STOPP/START criteria were first drafted in Ireland in 2008 and a new update in 2015 contains 80 STOPP and 34 START criteria arranged according to physiological system. It is accompanied by explanation as to why the prescription is potentially inappropriate based on the available evidence from guidelines and articles [3]. The criteria recognizes the dual nature of PIP by including a list of PIMs and PPOs [4–5].

Medications screened for appropriateness using STOPP/START criteria have been significantly associated with ADRs [6]. The PIPs were found to be linked with ADRs and medication non-adherence in older adults [7]. For example, Laroche et al, 2007 reported that ADR prevalence was higher (20.4%) among patients with inappropriate medication use in France [8]. A two fold increase in ADRs have also been reported among elderly people experiencing PIPs [9]. Apart from ADRs, PIPs may have cost implications. A systematic review designed to evaluate the economic impact of PIPs among elderly people suggested its economic burden was substantial [10].

The criteria has been applied in the context of various medical conditions, including cardiovascular disorders (CVDs). PIPs in the elderly people could negatively impact their clinical outcome due in part to, the complexity of the prescribed medications [11]. In addition to the complexity of cardiovascular medications, patients above 65 years of age have a dynamic physiology which can also increase risks associated with PIPs [12–13]. According to many studies the proportion of inappropriate medications in elderly chronic patients ranges from 25% to 50% [13–15].

In recent times, CVDs have emerged as major causes of hospital admissions in developing countries, including Ethiopia where they are among the leading non-communicable diseases [16]. Despite the growing evidence on multiple CVD admissions, measures to improve the quality of prescriptions are inadequate. In addition, factors implicated with prescription inappropriateness among patients hospitalized due to CVDs are not determined in the local setting. Hence, Understanding of the scale of the problem and contributing factors is essential in designing interventions to improve cardiovascular health. Therefore, this study aimed at assessing inappropriate prescribing and associated factors among elderly patients with cardiovascular disorders using the updated START/STOPP screening criteria.

## Patients and methods

### Study setting and period

The study was conducted at the medical ward of University of Gondar Hospital (UOGH) from 1 December 2016 to 30 May 2017. UOGH is a teaching and referral hospital that serves a catchment population of over 5 million people in the northwest Ethiopia. The medical ward has a 62 bed capacity and inpatient care is provided to cardiovascular patients including HF, AF, HTN, IHD and other CVDs.

### Study design and population

A hospital-based cross-sectional study was conducted among elderly patients with CVDs. Individuals aged 65 years or older who were admitted to a medical ward with either new or known diagnoses of CVDs were included. However, those with incomplete medication documentation were excluded.

### Operational definitions and definitions of terms

PIP indicates the use of medications in a situation where the risk of an adverse drug event (ADE) outweighs the clinical benefit and in the omission of clinically indicated medications in the absence of contraindication [1]. PPO refers to medications that should have been initiated in the elderly CVPs according to the START criteria [2]. PIMs are medications that shouldn’t be given for older CVPs according to the STOPP criteria [2].

### Data collection methods

A structured questionnaire containing 23 items was adopted from the STOPP/START criteria [3]. Fifteen questions were taken from the STOPP criteria. The remaining eight were prepared from START criteria. The STOPP/START criteria contains 114 items, of which 32 were cardiovascular and coagulation disorder related criteria. But, due to lack of availability of medications in the study setting the criteria were reduced Into 23 items. The questionnaire contained socio-demographic data, patient medication experience, and type of diagnosis, hospital stay, and number of medications during hospital stay and at discharge. Patients were consecutively recruited and data were collected by two trained clinical pharmacists. The study subjects were evaluated at the time of admission to the medical ward of UOGH. Information on socio-demographic variables and current medication was retrieved from patients’ medical card. Patients were interviewed regarding (procurement of prescribed medication, compliance and over-the-counter drug use) for completeness of medication history. Each medication indicated for CVDs was subjected to STOPP and START criteria for its appropriateness and the number of PIPs were documented on a daily basis until the patient is discharged.

### Data quality control technique

The reliability (psychometric property) of the tool was evaluated and demonstrated a Cronbach alpha value of 0.879. The content of the questionnaire was reviewed by senior experts who has published research work using the STOPP/START tool. The tool was adopted from the validated standard criteria which was last updated in 2015 team of experts which made the questionnaire more reliable. A part of the questionnaire administered to patients was translated in to Amharic to maintain increased understandability and avoid bias. Prior to data collection, intensive training was provided to data collectors on contents of the questionnaire, data collection methods and ethical concerns. The questionnaire was pre-tested on 20 hospitalized patient prior to the actual data collection. Questionnaires filled out were checked by the principal investigator on a daily basis to ascertain completeness.

### Data analysis

All the statistical data were carried out using Statistical Package for Social Sciences (SPSS), version 20 (SPSS Inc., Cary, NC, USA). Descriptive statistics was presented using means with standard deviation (±SDs) and percentages (%). Bivariate analysis was applied to investigate the relationship independent variables, such as socio-demographic characteristics, number of medications and length of hospital stay have with the extent of PIPs. Binary logistic regression was employed to determine factors associated with PIP. One way AONVA was carried out to test the significance differences between diagnoses in terms of PIPs. The cutoff point for P-values was kept <0.05 with 95% confidence interval was employed for test of statistical significance.

### Ethical considerations

The study was conducted after securing ethical clearance letter from research and ethics review committee of School of Pharmacy, College of Medicine and Health sciences, University of Gondar. The clinical director of UOGH and medical ward coordinators were aware of the study and they permitted to conduct the study. Informed written consent was obtained from each patient prior to participation by informing the aim of the study. Patients were allowed to withdraw participation at any time during the data collection process. The prescribers were communicated to resolve the identified PIPs.

## Results

### Socio-demographic and clinical characteristics of patients

A total of two hundred and fifty patients were admitted to the medical ward during the study period. Of these, two hundred and thirty-nine patients who fulfilled the inclusion criteria, were included in the final analysis. The mean age of the respondents was 72.52 ±7.7 and nearly half were males, 123 (51.5%). More than half of the patients were urban residents, 127 (53.1%). Regarding cardiovascular disorders, more than one-third of patients were diagnosed with HF, 88 (36.82%). Nearly half of the patients (118 (48.59%)) had comorbidities, the most prominent one being respiratory disorders, 32 (27.12%). The mean period of hospital stay of the patients was 16.99±4.51. The overall mean number of medications prescribed during hospital stay was 3.97±1.55 it was 2.91±0.99 at discharge (Table 1).

**Table 1:** Sociodemographic and clinical characteristics of respondents 2016/2017 (n = 239).

Variable	Value
Age (mean ±SD)	72.52 ±7.7
Sex(males)	123 (51.50)
Residence (urban)	127 (53.10)
Comorbidities (n = 118)	
Respiratory disorders	32 (27.12)
Rheumatic and cardio-myopathy	25 (20.34)
Kidney disease	24 (21.19)
Thyroid disorder	19 (16.10)
Diabetes	18 (15.25)
Diagnosis (n = 239)	
HTN+ Stroke	69(28.87)
Heart failure	88(36.82)
Atrial fibrillation	13(5.44)
Peripheral arterial disease	19(7.95)
DVT	11(4.60)
HTN	19(7.95)
AF +stroke	8(3.35)
AF+CHF	12(5.02)
Hospital stay(mean ±SD)	16.99±4.51
number of medications during hospital stay(mean ±SD)	3.97±1.55
number of medications at discharge (mean ±SD)	2.91±0.99
Procured medications (yes)	239(100)
Taking medication (yes)	239(100)
OTC medication (no)	239(100)

Abbreviation: AF, Atrial fibrillation; CHF, Congestive heart failure; DVT, Deep vein thrombosis; HPN, Hypertension; OTC, Over the counter medication; SD, Standard deviation

### Prevalence of PIPs

The prevalence of PIPs in the ward was 61.5% out of the total 239 patients included in the analysis. The prevalence of one PIP was 83 (56.4%) while that of two and three IPs were 52(35.4%) and 12(8.2%), respectively. Overall, 221 PIPs were found the 115(52%) being PPOs and the remaining being PIMs. According to the STOPP criteria, prescription of anti-platelets with VKA in patients with chronic atrial fibrillation 31 (14%) was the most prevalent PIM followed by the prescription of any medication without any indication 39 (17.6%). Among medications prescribed without indication digoxin, 21(9.5%) was frequently prescribed followed by amlodipine 5 (2.3%). The START criteria retrieved omission of ACEIs in systolic heart failure/coronary artery disease as the commonly observed PPO 32(14.5%) followed by under prescription of appropriate beta-blocker in patients with stable systolic HF 30(13.1%) (Table 2).

**Table 2:** IPs based on individual STOPS/START criteria among older CVPs in 2016/2017 (n = 221).

PIPs	Frequency n (%)
**PIMs**	106(48%)
Any medication prescribed without any indication	28(12.7%)
Digoxin	21(9.5)
Unfractionated heparin	3(1.4)
Amlodipine	5(2.3)
Any medication prescribed beyond the recommended duration	0(0.00)
Any duplicate medication	2(0.9)
Digoxin for heart failure with normal systolic ventricular function	3(1.4)
verapamil or diltiazem with class three or four heart failure	0(0.00)
Beta-blocker with verapamil or diltiazem	0(0.00)
beta blocker with bradycardia	5(2.3)
Loop diuretics with dependent ankle edema	5(2.3)
Loop diuretic for treatment of hypertension in urinary incontinence	3(1.4)
Loop diuretic as first-line treatment for hypertension	0(0.00)
Thiazide diuretic with current electrolyte imbalance	0(0.00)
Aspirin in peptic ulcer patients without anti-acid	9(4.1)
Spironolactone with K-sparing medications without checking the level of K^+^	20(9)
Aspirin with VKA in patients with chronic atrial fibrillation	31(14)
ACEIs or Angiotensin Receptor Blockers in patients with hyper-Kalema.	0(0.00)
PPOs	115(52%)
VKA or direct thrombin inhibitors in the presence of chronic AF	12(10.43)
ACEIs with systolic heart failure/coronary artery disease	32(14.5)
Aspirin in the presence of chronic atrial fibrillation.	0(0.00)
Antiplatelet to coronary, cerebral or peripheral vascular disease.	5(2.3)
Antihypertensive therapy where BP> 160/90mmHg	29(12.15)
Statin therapy with coronary, cerebral or peripheral vascular disease	7(3.16)
Beta-blocker with ischemic heart disease	0(0.00)
Appropriate beta-blocker with stable systolic heart failure	30(13.1)
Total IPs	221 (100%)

### Frequently prescribed inappropriate medications

**Fig 1:**
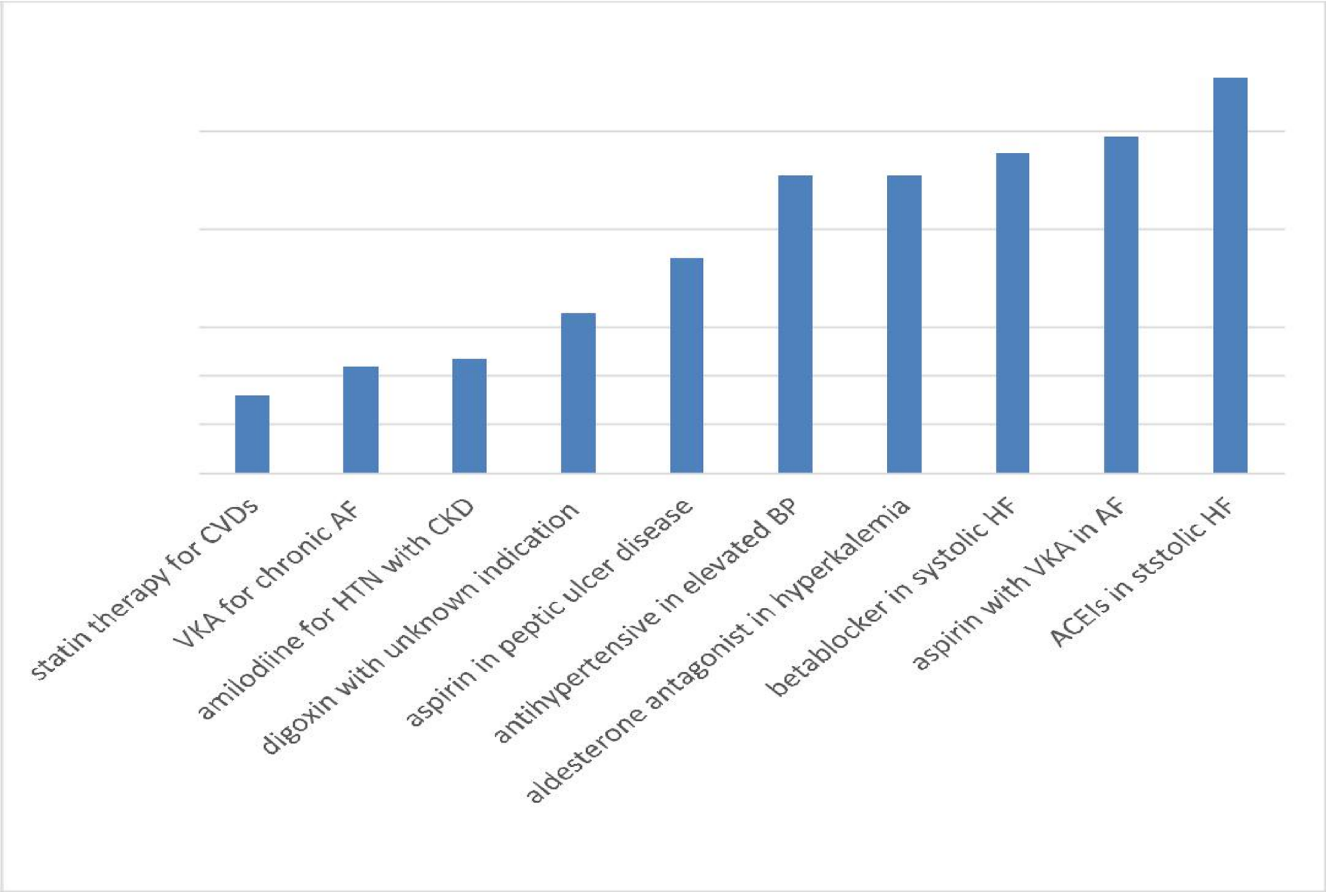
The top ten common drugs associated with IP among CVPs admitted to UOGH, 2016/2017.

ACEIs were the most commonly prescribed PIMs (15.6%) followed by the combination of aspirin with VKA (14%). Whereas, statin therapy in CVDs (3.16%) have been placed tenth under this list (Fig 1).

### IPs among different diagnoses

One way ANOVA test showed significant differences on the mean number of IPs per individual diagnosis *(f = 5*.*718*, *p<0*.*001)*. Post hoc analysis test indicated that the variations were particularly observed between hypertension (1.68±1.416) and deep venous thrombosis (DVT) (0.09±0.302, P = 0.004), hypertension and peripheral arterial disease (PAD) (0.32±0.478, P = 0.004), heart failure and DVT (1.48±1.134, P = 0.003), heart failure and PAD (*p = 0*.*001*), heart failure with stroke and DVT (1.17±1.111, P = 0.001), heart failure with stroke and PAD *(P = 0*.*001)* (Table 3).

**Table 3:** IPs in elderly CVPs presented with different diagnosis at UOGH 2016/17: One way ANOVA test.

Diagnosis	Mean(SD)	F-test	P-value
HTN	1.68±1.416	5.718	<0.001
AF+ stroke	1.63±0.518		
HF	1.48±1.134		
AF	1.08±1.188		
DVT	0.09±0.302		
PAD	0.32±0.478		
HF+ stroke	1.17±1.111		

### Factors associated with IPs

Binary logistic regression was undertaken to find out factors that affect the incidence of IPs. Accordingly, the binary logistic regression test showed that the incidence of IPs slightly increase for each day increase in hospital stay; AOR: 1.086 [1.016–1.160]. Every single medication addition at discharge expose patients for IP nearly two times: AOR: 1.924 [1.217–3.041]. In addition, the presence of comorbidity increased the likelihood of IP more than three times; AOR: 3.127[1.706–5.733]. However, other variables didn’t show any correlation with the incidence of IPs on binary logistic regression (Table 4).

**Table 4:** Factors affecting IPs in elderly CVPS attending UOGH 2016/2017.

Variables	IP status of patients		COR [95% CI]	AOR [95% CI]	
	Yes (147)	No (92)			
Age (mean ±SD)	72.26±7.583	72.95 ±7.907	0.989[0.956–1.022]	1.011[0.970–1.050]	
Sex					
Females	65(27.2)	51(21.3)	1	1	
Males	82 (34.3)	41(17.2)	0.637[0.377–1.077]	0.598	[0.333–1.075]
Hospital stay(mean ±SD)	15.77 ±4.111	17.26 ±4.856	1.11[1.041–1.180]*	1.086	[1.016–1.160]*
Comorbidities					
Yes	87(36.4)	31(13)	2.853[1.657–4.912]*	3.127[1.706–5.733]**	
No	60(25.1)	61(25.5)	1		1
number of medications during	3.13±0.909	2.83±1.145	1.378[1.149–1.652]*	0.751	[0.482–1.170]
hospital stay					
number of medications at	3.10 ±1.12	2.61±0.87	1.662[1.258–2.196]*	1.924	[1.217–3.041]**
discharge					

*Significant at <0.05

**significant at <0.01

## Discussion

Potentially inappropriate prescribing implies when there is utilization of medications in a situation in which the risk of an adverse drug event (ADE) outweighs the clinical benefit, and the omission of clinically indicated medications without known contraindication in patients with significant life expectancy [17]. Identification of the type of PIPs and the estimation of its magnitude would help to design interventions to reduce the impact of PIPs in patients’ quality of life. The STOPP and START tool is an explicit criteria to estimate the prevalence of IPs in older patents.

The present study sought to determine the prevalence of PIPs among elderly patients hospitalized with cardiovascular disorders using this tool. It was found that significant number of patients encountered PIPs (147 out of 239 admissions), which led the prevalence of PIPs in the set-up, nearly sixty percent (61.5%) and a total number of 221 PIPs. This figure is by far bigger as compared to Irish population (14.6%) [13], a retrospective study in Italy (28.6%) [18] and nearly 25% in India [19]. This discrepancy might be due to retrospective study in Italy, use different population other than CVPs in Irish and outpatient cardiac patients in India. But, comparable evidence obtained in CRIME study in which more than one-half of the participants were prescribed with PIPs [20]. Our study has also discovered that frequency of one, two and three IPs were 83(56.4%), and 52(35.4%), 12(8.2%) respectively. Napolitano et al identified the proportion of one IP to be only in one-third of patients but their study applied Beers criteria [15]. The proportions of one, two and three IPs were 24%, 6% and 2%, respectively in Irish older individuals [21].

In the present study, 106 (48%) PIMs occurred while 41% and more than one-half were reported by studies among geriatric Israeli patients and in Belgium respectively [17, 22]. The initiation of empirical therapy prior to the confirmation of the right diagnosis with objective investigations could contribute to higher prevalence of PIMs in CVPs. For example, initiation of digoxin before the estimation of cardiac ejection fraction in heart failure patients contributed to the occurrence of more IPs at admission because digoxin use is considered inappropriate if patients had PrEf or if they had no AF [5, 23–25]. Inappropriate use of digoxin was common (37%) among HF patients in United States since electrocardiography and echocardiography were not regularly performed [23].

In addition, prescription of antiplatelet along with anticoagulants in case of bedridden stroke patients, 31(14%) who complained for peptic ulcer 19(8.8%), was identified as PIM. A retrospective study in the same set-up has also demonstrated high proportion 36(51.4%) of inappropriate anticoagulants use[26]. Concomitant use of oral anticoagulants and aspirin was a common scenario amongst AF patients in Japanese elderly patients, which was associated with significantly increased risk for bleeding events [27].The increased in the consumption of anticoagulants could escalate the tendency of GI-bleeding due to complex pharmacology of these medications and subsequent exposure for chronic thrombo-prophylaxis [28–32].

Our study also found out that aldosterone antagonists were inappropriately combined with potassium sparing agent in 20 (9%) cases. Another study showed an increase in the risk of hyperkalemia due the introduction of aldosterone antagonists and ACEIs [33–34] their concomitant use is not recommended unless serum level of potassium is not monitored [33, 35]. The current study also found that a significant number of medications (28, 12.7%) were prescribed with no explanation of their indications. Those medications include amlodipine 5(2.3%) for CVPs in spite of the presence of more safe and effective alternatives. Generally, the use of calcium channel blocker (amilodipine) didn’t show a better outcome in CVDs including acute coronary disease among elderly Canadian patients [36].

Hospitalized patients admitted with CVDs usually require medications at discharge or throughout their life. These medications are considered important to reduce the progression and complications of the disease. But, omission of one or more essential medications led to 115 (52%) PPOs in our study based on the START criteria, ACEIs being the leading ones with 32 (14.5%) cases. ACEIs are routinely used for patients with systolic heart failure/coronary artery disease to prevent cardiac remodeling by inhibiting the activation of angiotensin pathway [37–41]. However, they are underutilized in these specific population despite their imminent role in maintaining cardiac function and regression of left ventricular hypertrophy. Underuse of ACEIs has also been reported among hospitalized elderly heart failure patients [42].

The omission of appropriate beta blockers in patients with stable HF has been observed in 30 (13.1%) of the patients. Only limited number of (21%) eligible myocardial infarction subjects received appropriate beta blockers in United States [43]. The prescription of beta blockers is determined by the presence of compelling indications such as hypertension, atrial fibrillation and ischemic heart disease and systolic heart failure [44–47]. Another medication among omitted ones was statin therapy as it was not initiated appropriately in 7 (3.16%) CVPs. A network meta-analysis indicated the prescription of statins in patients with a documented history of coronary, cerebral or peripheral vascular disease is advocated [48]. Another meta-analysis have shown superiority of statins in patients with high cardiovascular risk factors [49].

The current study demonstrated that significant number 29 (12.15%) of hypertensive patients received inadequate BP lowering agents. A much higher proportion, more than one-half, of older patients received inadequate antihypertensive in Spain [50]. The Irish Longitudinal Study reported that the most frequent medication omission was antihypertensive therapy where systolic blood pressure consistently above 160 mmHg (7). Our study has found that mean number of PIPs were higher among hypertensive (1.68±1.416, p: 0.004) patients as compared to other cardiovascular events. BP lowering agents are required both in the inpatient and outpatient set-up based on the level and the presence of comorbid conditions [51–52]. However, due to fear of organ hypo-perfusion, the initiation of antihypertensive might be delayed if eligible patients are not sufficiently stratified [51, 53]. Therefore, a delayed administration of anti-hypertensives exposed patients for target organ damage secondary to uncontrolled BP [54].

In the current study some predisposing factors were considered to be implicated with the frequency of IPs among CVS patients. Longer duration of hospital stay (LHS) was associated with the increased prevalence of IPs. Napolitano et al also discovered that the frequency of IPs in hospitalized elderly patients was affected by LHS [7, 55–56]. LHS increase the likelihood of hospital acquired disorders. The new diagnosis forced clinicians to use one more medications to manage hospital acquired diseases which in turn increase the number of medications prescribed and the probability of PIPs [22, 55, 57]. In addition, IPs might induce their frequency by increasing hospital stay because there is established evidence that IPs could be a reason for admission and prolong hospital stay [58–59]. Moreover, comorbidities have been found to increase the risk of PIPs in hospitalized CVPs in our set-up. Comorbidities such as constipation, osteoarthritis, recent history of fall and diabetes mellitus were found to be associated with PIPs in Japanese older population [60]. Comorbidities, including dysfunction of medication disposition organs usually require careful selection of medications and dose adjustment if necessary. But, these factors are usually not considered during initiation of medications for the elderly. The extent of IPs were significant among renal impaired Swedish [61] and Chinese elderly [62]. Overall, the culmination of estimation and discussions about PIMs direct our approach towards designing and implementation of interventional tools in order to minimize PIPs [2].

In general, the current study provided insight to the magnitude of PIPs in hospitalized elderly CVs patients in developing set-up. It enables us to pay attention to prescriptions for specific medications in CVPs. The prospective evaluation of IPs would increase the credibility of the evidence obtained from the study.

### Limitation of the study

Our study didn’t include qualitative evidences so as to view IPs from prescribers’ perspective. The sample size is also small to give representative and powered data on associated factors for IPs. In addition, factors might not be explicitly investigated in our study. It is also limited to single institution and on cardiovascular patients only.

## Conclusion

Potentially inappropriate prescription was higher among older cardiovascular patients. ACEIs were the most commonly mis-prescribed medications. Longer hospital stay, presence of comorbidities and prescription of large number of medications at discharge date have been found to be associated with the occurrence of IP. Periodic medication use evaluation should be implemented on these patients to improve the appropriateness of medications in the elderly CVPs. Factors such as comorbidities and polypharmacy should be taken into consideration as potential source of IPs while prescribing in the elderly CVPs. Furthermore, multicenter, powered/larger and qualitative studies that include the prescribers’ point of view is recommended.

## Abbreviations

ADE: Adverse Drug Event
ADR: Adverse Drug Reaction
ACEIs: Angiotensin Converting Enzyme Inhibitors
BBs: Beta Blockers
CVD: Cardiovascular Disease
CHARM: Candesartan in Heart Failure-Assessment of Reduction in Mortality and Morbidity study
CVPs: Cardiovascular Patients
CRIME: CRIteria to assess Appropriate Medication Use in Elderly Complex Patients study
DM: Diabetes Mellitus
DVT: Deep Vein Thrombosis
OP: Older People
UOGH: University of Gondar Hospital
HTN: Hypertension
HF: Heart Failure
IHD: Ischemic Heart Disease
IP: Inappropriate Prescription
LHS: Longer duration of Hospital Stay
ORBIT: the Outcomes Registry for Better Informed Treatment
PAD: Peripheral Arterial Diseases
PIMs: Potentially Inappropriate Medications
PIPs: Potentially Inappropriate Prescriptions
PrEf: Preserved Ejection fraction
PPOs: Potential Prescription Omissions
START: Screening Tool to Alert for Right Treatment
STOPP: Screening Tool of Older People Prescription
VKAs: Vitamin K Antagonists
